# Dimensions of professional competences for interventions towards sustainability

**DOI:** 10.1007/s11625-017-0439-z

**Published:** 2017-05-30

**Authors:** Francisca Perez Salgado, Dina Abbott, Gordon Wilson

**Affiliations:** 1 Faculty of Management, Science and Technology, Department of Science, Open University of the Netherlands, P.O.Box 2960, 6401 DL Heerlen, Netherlands; 2 College of Life and Natural Sciences (Geosciences), University of Derby, Kedleston Road, Derby, DE22 1GB UK; 3 Development Policy and Practice, Arts and Social Sciences Faculty, The Open University, Walton Hall, Milton Keynes, MK7 6AA UK

**Keywords:** Sustainability, Professionals, Practitioners, Competences, Skills, Action research, Interventions, Change

## Abstract

This paper investigates sustainability competences through the eyes of professional practitioners in the field of sustainability and presents empirical data that have been created using an action research approach. The design of the study consists of two workshops, in which professional practitioners in interaction with each other and the facilitators are invited to explore and reflect on the specific knowledge, skills, attitudes and behaviours necessary to conduct change processes successfully towards sustainability in a variety of business and professional contexts. The research focuses on the competences associated with these change processes to devise, propose and conduct appropriate interventions that address sustainability issues. Labelled ‘intervention competence’, this ability comprises an interlocking set of knowledge, skills, attitudes and behaviours that include: appreciating the importance of (trying to) reaching decisions or interventions; being able to learn from lived experience of practice and to connect such learning to one’s own scientific knowledge; being able to engage in political-strategic thinking, deliberations and actions, related to different perspectives; the ability for showing goal-oriented, adequate action; adopting and communicating ethical practices during the intervention process; being able to cope with the degree of complexity, and finally being able to translate stakeholder diversity into collectively produced interventions (actions) towards sustainability. Moreover, this competence has to be practised in contexts of competing values, non-technical interests and power relations. The article concludes with recommendations for future research and practice.

## Introduction

Sustainability issues are increasingly inspiring citizens, communities, organisations, professionals, businesses, governments, and international bodies to change their goals, practices and approaches. Since the emergence of ‘sustainability science’ (Kates et al. 2001), considerable research on its core questions has been conducted, finding and applying novel research strategies and methodologies. At present, it would appear that sustainability science is in transition from a descriptive to a more transformational mode, with new styles of research and epistemologies (Vilsmaier et al. 2015; Wittmayer and Schäpke 2014; Schneider and Rist 2014; Wiek et al. 2014; Benessia et al. 2012). These articles and their authors suggest that ‘actionable’ knowledge is produced during the transformational mode towards sustainability. This is what Nowotny (2003) calls ‘socially robust knowledge’, being the product of intensive and continuous interaction between science and society, with the knowledge production being process-oriented and often open-ended. ‘Actionable research’ thus generated, allows us to address the dynamics of the change processes towards more sustainable trajectories.

It is becoming evident that a whole new profession, that of the sustainability professional, is emerging within the hugely diverse and complex field of ‘sustainability’. On the one hand there is a big operating range, from managers, entrepreneurs, policymakers to consultants and accountants, to name just a few. On the other hand, there is a great variety of fields in which they work, for example, energy, water, climate change, government, urban design and planning, development, industry, business, engineering and technology, and many more. We are, therefore, in pressing need of bringing scientific knowledge and cohesion to this new area, and answering questions concerning the effectiveness of the work of sustainability professionals. For example, what knowledge and skills do they use in their day-to-day practices? What is their role in the navigation towards sustainability? What skills are necessary for implementing transformation? How do sustainability professionals become effective ‘change agents’, transition managers, problem solvers, and effective performers? What problems do they face?

The concept of ‘competence’ (or competency) has become popular since the 1990s in fields where an integration of different skills and knowledge domains is necessary (Van der Klink and Boon 2002; Salganik et al. 1999). Consequently, the scientific literature that refers to competences is large, covering a variety of fields. In this paper, we have had to be selective, restricting that cited mainly to literature which deals substantively with sustainability competences. Much of this literature pertains to HE and sustainability competences where we focus on key articles for comparative purposes (see below). That aside, however, our bias is towards sustainability competences in relation to sustainability practitioners, where the literature is less.

In addition to the scientific literature, the term ‘competence’ is widely and commonly used by practitioners themselves. The integration of knowledge domains and skills certainly applies to the sustainability field, and several researchers have investigated these competences, capabilities or skills for sustainability among professional practitioners and have done this from different perspectives (e.g. MacDonald and Shriberg 2016; Thomas et al. 2013; Willard et al. 2010; Hurlimann 2009; Martin 2005, 2008). These studies show and emphasise a diverse and complex set of knowledge and skills, but note that, except for Willard et al. (2010), the focus is not on sustainability professionals per se (the subject of our enquiry) but on a range of professionals who may have to grapple with sustainability issues (among others) in their work. Also, only Willard et al. (2010) and Martin (2005, 2008) focus on professional sustainability needs tout court, while the others have a further focus on meeting these needs through higher education (HE). Willard et al. report as key finding that soft skills needs are ‘communication with stakeholders’, ‘problem solving’ and ‘inspiring and motivating others’, while hard skills needs are ‘strategic planning’, ‘systems thinking’ and ‘project management’. In the grey literature, there is, for example, a worldwide UN-Accenture report showing the impact that sustainability will have on companies’ envisioning and how CEOs can lead the process towards a sustainable economy (Lacy et al. 2010).

Also in HE competences for sustainability have gained considerable attention. Recognising the burgeoning literature in the field and drawing on a systematic international review, Wiek et al. (2011) synthesised a sustainability competency framework which is supported by other scientists (Thomas and Day 2014; Barth et al. 2016). This framework consists of one comprehensive, over-arching competence ‘sustainability research and problem-solving competence’, which integrates five key competences. Barth et al. (2007) and Rieckmann (2012) also propose several key competences for sustainability, such as ‘competency in interdisciplinary work’ and ‘competency in self-motivation and motivating others’. We will address these findings later in more detail.

However, note that there is criticism in the conceptual paper by Mochizuki and Faveeda (2010) on efforts trying to reach universalism in the competence discourse. They state that “core competencies may not be always conceived as universal” (Mochizuki and Faveeda 2010: 395) and that differences could exist, for example between ‘developed’ and ‘developing’ countries. They mention that “competences have no meaning unless they are enacted in practice and connected to assessment in a particular context” (Mochizuki and Faveeda 2010: 400). Their critique matches the constructivist approach for developing competences proposed by Stoof et al. (2002). These authors state that the ‘people working with the competences’ should be one of the focal points in developing competences and they argue that contextualisation will generate diversity in competence definitions and development as a consequence. Using this constructivist approach Lansu et al. (2006) find the following key competencies for the professional level in environmental sciences in the context of sustainable development: diagnosis, research and intervention competence (Lansu et al. 2006). Pérez Salgado et al. (2012, 2014) further describe and develop this ‘intervention competence’ as the competence for the transition process towards sustainability in an international competence-based educational programme (Wilson et al. 2011).

In the presented research, we focus on the intervening part towards sustainability conducted by professional practitioners, and will not address other competences for sustainability such as ‘systems thinking’, ‘communication’, ‘multi-disciplinary analytical skills’, etc., although we acknowledge that inevitably there are overlaps between them where a comparative study could inform future research. At this juncture, however, we focus on the professionals: what abilities and skills do they say that they need for conducting the actual change process? We present empirical data obtained with sustainability professionals concerning this specific intervention competence, which focuses on the actual change process towards sustainability conducted or led by a professional. A key challenge within the change process is that most sustainability practitioners work with multiple stakeholders who normally are separated from one another in their daily lives and who have diverse experiences, interests and values. These other stakeholders may be sustainability professionals from different organisations with different areas of expertise. They may also be professionals from different sectors, politicians in national or local government, activists, and users of environmental services as businesses or members of the public. The ‘working with other stakeholders’ theme spans a diversity of:

- Sustainability concerns and sectors;
- Approaches, from disciplinary scientific studies whose primary purpose is to provide an evidence base to sustainability (solutions) in different contexts;
- Country locations, and
- Scales, from international agreements to national strategies to local implementation.

There exist challenging micro-dynamics of engaging and working with others, where much of the responsibility for positive engagement and outcomes lies with the various actors. Good and timely intervention cannot be guaranteed. In addressing these situations, the sustainability professional as one kind of actor typically has to:

- Help reach a decision for action that is acceptable to different stakeholders, even if it means setting aside, at least partially, one’s own perspective.
- Bring evidence to bear on the matter under investigation in order for their viewpoint to become an informed, and even a persuasive, opinion in the eyes of other stakeholders.
- Listen and apply their perspective to evidence that is supplied by others.
- Recognise that other perspectives might be at the extremes encountered normally, but none can be ignored totally and all have to be understood. It is important here to understand that ‘perspective’ includes, but cannot be reduced to, one’s disciplinary, scientific understanding of an environmental problem. Perspective also includes understanding that is associated with one’s ‘lived experience’. This is our evolving knowledge that derives from everyday practice, engagement with others, the nature of the problems that we have to address, and the values and behaviours that are informed by those of our work organisation and our personal socio-economic circumstances (Abbott and Wilson 2012, 2014, 2015).

Against this background, the authors explore the dynamics of necessary professional skills and behaviours that may lead change processes towards sustainability. In particular, the research focuses on the competences needed to successfully conduct the desired change processes. Thus our study investigates how sustainability professionals view key aspects of their work, and the skills and behaviours they feel that they need to perform well at it. It explores in particular the notion of ‘intervention competence’ as an agglomeration of the knowledge, skills, attitudes and behaviours that are required for sustainable solutions and decisions through working with others. We point out that it is important to distinguish between intervention and action, where the latter is the habitual act one performs without applying critical thinking. The authors designate intervention, however, as a consciously performed new act that has its starting point in conscious, critical and creative thinking and which (in the case of sustainability issues) takes place in a context of multi-actor engagement. It leads potentially to ‘new’, not previously displayed acts and trajectories towards sustainability, and to the key question of this paper:

- What are the specific dimensions of intervention competence that enable sustainability professionals to facilitate effective intervention towards sustainability in multi-stakeholder settings?

Following the above introduction to the study, the second section of the paper examines the literature on human engagement. The third section discusses the literature on sustainability professionals and explains the notion of intervention competence for sustainability. A methodology based on the principles of action research is described in the fourth section, together with its application and design for the workshops. Empirical results and what they tell us about intervention competence for sustainability professionals are covered in the fifth. The sixth section digs deeper and discusses the results in a broader context and from a reflexive perspective. This section probes the underlying reasons why the sustainability professionals formulate the dimensions of intervention competence in the way they do. The final section presents the conclusions, reviews the findings and raises further questions.

## The challenge for productive human engagement in multi-actor settings

A critical philosophical foundation for working with others lies in the work of Jürgen Habermas. To the fundamental question, ‘What makes us human’, he answers in terms of two capabilities (Edgar 2006: 62–64):

- Our ability to ‘labour’, by which Habermas means our ability to transform our physical environment or ‘nature’ for productive use, and
- Our ability to interact and communicate with each other, not just in the sense of conveying information, but in justifying our beliefs in the form of discussion, debate and challenge.

Habermas (1987) goes on to argue for human engagement to embrace ‘communicative action’ which has been defined as ‘free and open discussion of all relevant persons without any form of coercion’ (Edgar 2006: 23). In a slightly later work Habermas (1992: 88) amplifies what he calls the ‘ideal speech situation’. Applied to sustainability processes (or change), the ideal speech situation describes the deliberate attempt to create the conditions for free and transparent communication among all stakeholders in an intervention. Habermas posits ‘communicative action’ in opposition to ‘instrumental action’ which concerns getting work done by the most efficient and effective means, and which short-cuts the discursiveness of the former. While Habermas (1992, 2011) recognises the importance of instrumental action for many human exchanges (in a supermarket, in administration, at work and so on) he is concerned by the way that it builds upon itself. If unchecked, instrumental action ultimately overwhelms communicative action, thus limiting human engagement and narrowing the choices available in life, including work-life.

Also relevant to this paper is the work of Michel Foucault who examined the flow of knowledge claims through communication acts. Foucault argues that what comes to be constituted as accepted knowledge (‘the truth’) reflects power relations between the engaging actors (Foucault 1980). In addition, our paper draws on the concept of ‘situated knowledge’, introduced by Donna Haraway (1988). This concept explains the existence of diverse knowledges around the world, each embedded in their specific eco-cultural-social-gender-bodily reality.

The extent to which multi-stakeholder engagements of sustainability practitioners approach communicative rather than instrumental action and the extent to which the outcomes reflect dominant power relations in diverse situations form the philosophical backdrop of this paper.

## Professional competences for interventions towards sustainability

The competences for sustainability of professionals practitioners working in a variety of fields have been investigated by several researchers, who are discussed here with respect to the focus of this paper. We proceed by addressing intervention competence and generate a working definition at the end of the section.

Thus, regarding the literature on skills, competences and capabilities in sustainability professions, MacDonald and Shriberg (2016) analysed sustainability leadership programmes from alumni perspectives. They stress the importance of incorporating in leadership programmes practice-oriented skills that promote productive engagement as outlined in the previous section. Skills include, for example, negotiation, coalition building and facilitation for sustainability leaders. The generic skills and capabilities that (Australian) employers would like to see in graduates are documented by Thomas et al. (2013), reporting skills in listening, oral and written communication, and negotiating and reasoning, and capabilities in willingness to learn, teamwork, ethical values and attitude, adaptive behaviour, reflecting on experiences and inclusive perspective. These skills and capabilities add a learning dimension to the purpose of productive engagement. As they are generic, however, such skills and capabilities are not confined to the sustainability field. Indeed, one could argue that they apply to any professionals who have to work in multi-stakeholder contexts. Thomas et al. (2013) also hone in specifically on capabilities for sustainability, where communication, critical thinking, decision-making, reflecting on experiences, and holistic and systems thinking are all of high importance.

The theme of productive engagement is continued by Willard et al. (2010) who conducted a broad survey specifically among sustainability professionals (80% from Northern America) to establish criteria and competences for practice. They found that the following ‘soft skills’ are the most important: communication with internal and external stakeholders, problem solving, and inspiring and motivating others. Strategic planning is mentioned as the most important ‘hard skill’. At a more generic level and from a systems perspective, Martin (2005, 2008) reported that future practitioner qualifications should include the ability to manage change and conflict, and problem-solving in a non-reductionist manner for highly complex real-life problems, but he also recognised the importance of action learning, dialogue, inquiry, participation and inter-professional partnerships.

This apparent consensus for skills, capabilities and ultimately competences in productive engagement and associated learning among professionals is disrupted, however, by Hurlimann’s (2009) survey of Australian urban, planning professionals with respect to environmental skill areas that should be part of HE curricula. Of the 49 respondents, six prioritised ‘community consultation’, three ‘broadened perspectives’, two ‘communication’, two ‘sensitivity’ and one ‘empathy’. These low numbers indicate that in this long-standing, established field, the need for productive engagement skills is not seen as a priority for most professionals. In fact, the highest priorities were accorded to critical thinking (13 respondents) and independent inquiry (8 respondents), which are standard generic key skills outcomes of HE curricula whatever the subject and are intended to equip students for future lives as both citizens and in the work place.

Much of the HE literature that relates to sustainability competences is less concerned directly with professional acquisition, however, and more with students in general—who may or may not become professionals who have to deal with sustainability issues. Some of this literature focuses on the process by which these competences may be acquired through HE study, others on the nature of the competences themselves, and a few on both aspects.

Thus, the focus for Barth et al. (2007) is on how competences are acquired through a learning process. Other than as a starting point for such a learning process, they are less concerned with what these competences are, although their final discussion refers to interdisciplinary collaboration and the ability to motivate others (alongside planning and implementation skills) in line with the general emphasis on productive engagement noted above.

Rieckmann (2012) conducted a Delphi study of experts from Europe and South America who selected and ranked 19 competences that are critical for sustainability and which should be developed by HE. The top rank was accorded to ‘systemic thinking and handling of complexity’, while in total ten competences related to productive engagement directly. The experts also confirmed the importance of HE for sustainable development, although this is not surprising given that over half (42/70) were scientists who had published work in the area.

As stated in the introduction, Wiek et al. (2016, 2011) synthesised a sustainability competency framework which is supported by other scientists (Thomas and Day 2014; Barth et al. 2016). This framework consists of one comprehensive, over-arching competence ‘sustainability research and problem-solving competence’, which integrates five key competences. Two of these—systems-thinking competence and interpersonal competence—relate obviously to productive engagement between stakeholders, but, interestingly, definitions of the other three all require the ‘ability to *collectively*’ (emphasis added) do something: analyse, evaluate, and craft rich ‘pictures’ of the future (anticipatory competence); map, specify, apply, reconcile, and negotiate sustainability values, principles, goals, and targets (normative competence); design and implement interventions, transitions and transformative governance strategies towards sustainability (strategic competence). In short, all five are based on the assumption of productive engagement between stakeholders, or, as Wiek et al. (2011) put it: ‘All rely on collaborative approaches to create ownership’.

Stoof et al. (2002) state that the actual context of the professional practitioner is a crucial aspect in developing professional competences. Critically comparing several methods, a qualitative approach in which there is interaction with and between professionals seems to be the most appropriate for identifying competences in new fields (Van der Klink and Boon 2002). This approach was applied in domains such as Human Resource Management, Health, Education and Economics, Business and Public Administration (Van der Klink and Boon 2002; Van der Klink et al. 2007; Boon et al. 2013), resulting in different sets of competences for each domain. Using this approach intervention competence was identified as a key competence for environmental scientists in the context of sustainable development (Lansu et al. 2006). This intervention competence was also part of an innovative, open resource international programme on sustainability (Wilson et al. 2011) in which students, practitioners and citizens interacted with each other. The development of intervention competence in this programme involved the following elements (Pérez Salgado et al. 2012, 2014):

- Appreciating the importance of (trying to) reach decisions or interventions;
- Being aware of a multitude of solutions, related to different perspectives and to different stakeholders;
- Being able to engage in political-strategic thinking, combined with personal goal-directedness (strategic decision making);
- Being able to steer towards collectively produced proposals and decisions, articulating policies and/or proposing initiatives which challenge existing non-sustainable practices;
- Being able to translate this diversity into propositions and decisions for interventions.

These authors state that this competence should be further conceptualised and investigated, specifically in interaction with practitioners, in order to gain a deeper insight into the intervention process itself. Additional questions that arise are: to what extent do sustainability practitioners recognise these elements or dimensions? What other dimensions do they suggest? With what competences do they consider that they should really be equipped?

In the following sections we try to answer these questions using new empirical data. We end this section by presenting the definition of intervention competence for sustainability that is used in the research and communicated and discussed with the professional practitioners:

‘the combination of knowledge, skills, behaviours and attitudes that enable a person to devise, in a process of consultation with relevant stakeholders, one or several solution(s) or decisions for a sustainability issue and subsequently successfully conduct the change process towards sustainability’.

## Methodology

Four articles cited in the previous section feature practitioner perspectives, whilst the more voluminous literature concerns students in HE. Three of these articles, however, represent practitioners through student alumni (MacDonald and Shriberg 2016), employers (Thomas et al. 2013) and specifically a systems disciplinary perspective (Martin 2005, 2008).

Only Willard et al. (2010) draw directly from sustainability professionals themselves through a broad survey. The results are interesting and enable some generalisations to be made about sustainability competences. However, as Van der Klink and Boon (2002) point out, data from ‘surveys are not of great value—due to the nature of the research method—in developing a view on the distinctive qualitative structure of competencies within a profession’. In other words, survey data alone, even when containing open-ended questions, cannot deliver the dialogue and probing that is essential for a fuller understanding. Thus, a qualitative, discursive approach is required, at least to complement the survey.

With respect to our work, the research question required us to grasp the specific dimensions—that may be used both explicitly and tacitly—to facilitate effective intervention for sustainability in varied, complex, multi-actor settings. Thus, as with Willard et al. (2010), the starting point for our inquiry was the practitioners themselves, but we also wanted them to develop their own ideas about competences through their experiences of intervention and the sense that they make of these. Methodologically, this required direct engagement with the lived experiences of practitioners, their professionalism and ways of intervening.

This is why an action-research approach (McNiff 2013; McNiff and Whitehead 2011; Kemmis 2010; Van der Klink and Boon 2002) involving both researchers and practitioner participants in dialogue was considered to be the most appropriate methodology for our study. Only as a result of such engagement were we able to draw out individual in-depth knowledge of how to organise change within specific and differing work contexts, and individual organisational, resource and other constraints.

A critical point for the methodological choice is that an action research approach empowers practitioners through their becoming active in the research processes. Through their participation they create new insights and contribute to innovative ideas and theories based on their own lived experiences and instincts. Their insights feed into everyday practice and policy-making, and into theoretical discourse and critical analysis in the world of academia via the scientists who take an action research approach. In turn, this develops a new form of scholarship (Wittmayer and Schäpke 2014; McNiff and Whitehead 2011; McNiff 2013; Wiek et al. 2012a, b; Benessia et al. 2012; Kemmis 2010). As described in the section “Professional competences for interventions towards sustainability”, this method has been applied successfully in the fields of Human Resource Management, Education and Economics, Business and Public Administration.

Our empirical research centred on two semi-structured practitioner workshops where knowledge was co-produced through discursive processes. As befits an action research approach, we sought in these workshops to meet Guba and Lincoln’s (1989: 245) four criteria for authenticity: (1) ontological authenticity in that both practitioners and researchers became better informed about themselves through their participation; (2) educative authenticity in that they gained enhanced understanding of each other (among participants and between participants and researchers) through the act of participation; (3) catalytic authenticity in that a senior member of the Dutch Association of Environmental and Sustainability Professionals (Vereniging van Milieuprofessionals—VVM) endorsed the research and was, alongside the university researchers, a facilitator in the workshops; and (4) tactical authenticity in that workshop participants were empowered to act through the VVM. Drawing on Kemmis (2010), who was one of the first to apply action research to sustainability issues, we also aimed to contribute to the evolution of professional practice for which its practitioners are not just accredited operatives, but also stewards.

As with all qualitative research, an action research approach cannot escape issues of rigour, trustworthiness and reliability of results (Dick 1999). One approach to addressing such issues is to attempt a transfer of the notion of replicability that is found in quantitative and experimental, positivist research to qualitative research by systematic coding—that is, deriving and developing concepts from the raw qualitative data (Corbin and Strauss 2008: 65, 159). Because our research, however, was based on discursive and hermeneutic processes of knowledge construction, we chose an alternative phenomenological and constructionist approach (Guba and Lincoln 1989: 8; Mohan and Wilson 2005). Here, rigour, trustworthiness and reliability are not signified by replicability, but through a hermeneutic process of convergence between diverse stakeholders.

In practice this meant that our practitioner participants were from a variety of organisational settings, holding in common their membership of VVM, who came together on a joint project, a large part of which involved reflecting critically with one another and with propositions put forward by the researchers. This high degree of stakeholder participation and critical reflection among the practitioners and between practitioners and researchers provided key sources of rigour, requiring at every stage the reconciliation of multiple views and giving opportunities to discuss and correct misconceptions (Dick 2007). The process was enacted during the course of the first workshop, through the opportunities provided for the practitioners and three researchers to engage in the final plenary phase of the first workshop, through the production of the report of this workshop, and through the discussion of this report’s findings at the second workshop (Steps 3–5 below).

With respect to data and results generated, the process of engagement and critical reflection extracted categories and the patterns that they made through their overlaps and linkages, initially by the practitioners from personal stories they told, and later through mapping onto the five dimensions of intervention competence outlined in the previous section of the paper (see Gläser and Laudel 2013 for a description and analysis of this approach). In detail, the steps were:

1. The research process that lasted from November 2013 to July 2014 and ran two participant workshops (Workshop 1 and Workshop 2). For Workshop 1, an open invitation for participation was made to VVM members in English. The invitation stated that the broad aim of the half-day workshop was for professionals to identify for themselves, through discussion with each other, the skills they require for successful interventions. A central location was chosen for relative ease of access.
  Seventeen members accepted the invitation.^1^ These comprised an almost equal number of men and women participants of differing nationalities, aged between 25 and 60, representing private companies, public institutions, non-governmental organisations (NGOs) and some entrepreneurs (working for themselves). Sectors and policy interests were diverse, and included energy, municipality-led climate change intervention, provincial sustainability, information technology, health, international environmental change, national development policy and the green economy. Some of the professionals were new at their jobs whilst others were highly experienced. They had a variety of roles and responsibilities such as public relations specialists for sustainability, (senior) sustainability consultants, sustainability project leaders and a company director. The workshop was to be conducted in English in order to accommodate all participants and facilitators.
2. Those who accepted the invitation were sent a further communication requesting them to reflect and make personal notes, to bring to Workshop 1, on their experience of a specific work situation which had required them to engage with diverse stakeholders from other external organisations, including professionals, politicians or the general public. They could choose successful or unsuccessful interventions, or anything in between.
3. Following a brief introduction, Workshop 1 was facilitated by three researchers—the authors of this article—and the senior VVM staff member. Their role was strictly facilitation as participants shared and discussed in the first phase their notes of personal experiences in four groups, illustrating the rich variety of working with a diverse range of stakeholders. The second phase was where each group compared their experiences and findings, looking for ways in which they were similar and contrasting. This led to the groups conceptualising and categorising key knowledge, skills, attitudes and behaviours that are required for successful intervention. Each group then presented its findings at the third phase, the plenary session. A comparison of each group’s findings led to further refinement of the key conceptual categories (knowledge, skills, attitudes and behaviours) that they had identified for successful interventions and the organisational and contextual aspects which enabled or constrained specific applications. In summary, workshop 1 took a primarily inductive approach where conceptual categories were not created in advance, but firstly by practitioners themselves on examination and comparison of their personal experiences.
4. A post-workshop review undertaken by the researchers comprised a more deductive approach where we attempted to map categories developed by the practitioners onto the five dimensions of intervention competence that had been generated through earlier research and which we have presented in the section on competences above. A draft report was then prepared and sent to participants for validation. This allowed each participant to analyse and comment individually on the workshop findings as we had represented them, with the feedback being incorporated into a second version. This process of reflection on workshop 1 resulted in modifications to the five dimensions, reducing the number to three, and also added four further dimensions of intervention (see “Results” section below).
5. Workshop 2 took place 7 months later, as part of the National Environment Day 2014, organised by the VVM. The workshop was publicised by the VVM and participants were invited to sign up. Ten professionals participated, two of whom had attended Workshop 1, the rest being a different set of participants. The group was similarly diverse (gender, age, professional maturity and field). Workshop 2 was facilitated by the same three researchers and the senior VVM staff member in a similar mode to that of Workshop 1. It used the second report of Workshop 1 as its starting point, and participants were invited in sub-groups to both validate and engage critically with its findings. Consequently, we could re-visit intervention competence and its dimensions in a continuous, reiterative cycle of action and reflection. In this way, Workshop 2 served both to corroborate the findings of Workshop 1 and to provide deeper perceptions and insights, as well as challenges, concerning intervention competence in practice. The results were sent to all participants (from Workshop 1 and 2) for comments which were used for the final version. The insights from Workshop 2 support the more explorative section “Discussion: the search for further meaning in the results” and the final section “Conclusions” below.

Finally, this mode of research suggests that there is a strong argument that diversity of experiences and values can be a source of social learning (Wilson 2009). This is precisely what we attempted in the two workshops, drawing on the practical and experiential knowledge of a diverse range of sustainability professionals.

## Results

Table 1 comprises a slightly edited (for reasons of conciseness only) version of the final flip-charts of each group in the first workshop. The knowledge, skills and behaviours in each column are in the order in which the participants presented them. The table provides, therefore, the raw data for our results.

**Table 1:** Knowledge, skills, attitudes and behaviours associated with intervention competence, as identified by sustainability professionals

Group 1	Group 2	Group 3	Group 4
• Build bridges • Have sympathy for all stakeholders • Be transparent • Have and show confidence throughout the process • Being inviting • Use effective mass-media • Distribute tips for the change/intervention • Appeal to ‘what’s in it for me’ for the stakeholders	• Participate in dialogue • Be transparent • Have overview of the discussion • Be proactive; showing anticipation pays off in a positive way • Be explicit about rewards for the different stakeholders • Be able to connect worlds, to add the social dimension to economic gain	• Variety of views leads to creativity • Learning = growing • Find similarities through speaking of the personal • Take broader perspective on solutions • Generate buy-in • Build trust • Align conflicting interests • Combine different aims and scopes • Find common ground, maybe by paying attention to value(s) • Be aware of existence of hidden agendas • Be aware of cultural differences • Be aware of diverse organisational structures • How to maintain vitality?	• Build trust • Build confidence • Build understanding • Have good personal knowledge base • Show drive • Maintain focus • Accept different viewpoints • Be inventive • Take care that the stake-holders get to know each other formally and informally • Design a collective road map to the future • Show and practise perseverance • Be aware of time constraints of stakeholders

Key, overlapping and sometimes recurring words and phrases in these flip-chart presentations were used by the participants to describe these skills, attitudes and behaviours. They include patience, perseverance, building trust, showing and building confidence, finding common ground, building bridges, building on similarities, understanding different organisational and institutional cultures, ability to align competing interests, revealing hidden agendas and creating road maps. Underpinning knowledge was represented by phrases such as: distributing tips for change/intervention, having an overview of the discussion, learning = growing, and having a good personal knowledge base.

These words and phrases, therefore, appear to be what sustainability professionals consider to be the building bricks of intervention competence. Overall, they indicate that intervention competence requires attention to process, to developing a means of understanding and promoting transparency in communicating different agendas.

During the post-workshop 1 discussion (Step 4 in the “Methodology” section), the researchers noted that several of the aspects in Table 1 concern productive engagement between stakeholders, reflecting the skills/capabilities/competences for sustainability that were consistently reported in our literature review in the section “Professional competences for interventions towards sustainability”. Importantly for our research, they also underlie the elements or dimensions of intervention competence that were reported in that section. Thus, these earlier tentative dimensions were corroborated by the practitioners in the workshop.

A further reflection on Table 1 and these dimensions, however, showed us that some of the latter overlap. As a result, we combined the following dimensions into two, because of their close inter-relationships: (1) ‘the awareness of a multitude of solutions’ with ‘the engagement in political-strategical thinking’; (2) ‘the ability to steer towards collectively produced proposals and decisions’ and ‘the ability to translate this diversity into propositions for interventions’. Also, the exact phrasing of the dimensions has been refined and sometimes expanded under influence of the new empirical data. Moreover, the Table 1 data suggest four further dimensions that give a more complete view of the intervention process. Thus, the following offers an expanded and adapted list of seven dimensions for intervention competence, arising from the data generated by the first workshop, a list that was confirmed by the second workshop (* denotes a new dimension while the others are extensions/refinements of the original dimensions; indented are examples from the practitioners from Table 1):

- Being able to appreciate the importance of (trying to) reaching decisions or interventions, connected to a motivation to act.
  Example from Group 1, ‘Distribute tips for the change/intervention’; Group 2 ‘Be explicit about rewards for the different stakeholders’; Group 3, ‘Maintain vitality’; ‘Group 4, ‘Show drive’, ‘Maintain focus’.
* Being able to learn from lived experience of practice, and connecting it to one’s own scientific knowledge.
  Examples from Group 3 ‘Learning = growing’; Find similarities through speaking of the personal’; Group 1 ‘Have and show confidence throughout the process’; Group 4 ‘Have good personal knowledge base’.
- Being able to engage in political-strategic thinking, deliberations and actions, related to multiple perspectives and actors, combined with personal goal-directedness.
  Examples from group 1 ‘Have sympathy for all stakeholders’; Group 2 ‘Have overview of the discussion’; ‘Participate in dialogue’; Group 3, ‘Take broader perspective on solutions’; Group 4, ‘Accept different viewpoints’.
* Being able to show goal-oriented, adequate action.
  Examples from Group 2 ‘Be proactive—showing anticipation pays off in a positive way’; Group 3 ‘Combine different aims and scopes’, ‘How to maintain vitality’; Group 4 ‘Design a collective road map to the future’, ‘Show drive’, ‘Maintain focus’.
* Being able to adopt and communicate ethical practices during the intervention process.
  Examples from Groups 1 and 2 ‘Be transparent’; Group 3: ‘Find common ground (..) by paying attention to value(s)’, ‘Groups 3 and 4 ‘Build trust’.
* Being able to cope with the degree of complexity. The complexity may refer to a multitude of aspects during the change process.
  Examples from Group 3 ‘Be aware of diverse organisational structures’; Group 4 ‘Be aware of time constraints of stakeholders’.
- Being able to steer stakeholder diversity into collectively produced propositions and decisions for interventions towards sustainability, articulating policies and/or proposing initiatives which challenge the existing non-sustainable practices, and are change-effective.
  Example from Group 1 ‘Build bridges’; Group 2 ‘Be able to connect worlds, to add the social dimension to economic gain’; Group 3 ‘A variety of views leads to creativity’; Group 1 ‘Distribute tips for the change/intervention’.

**Fig. 1:**
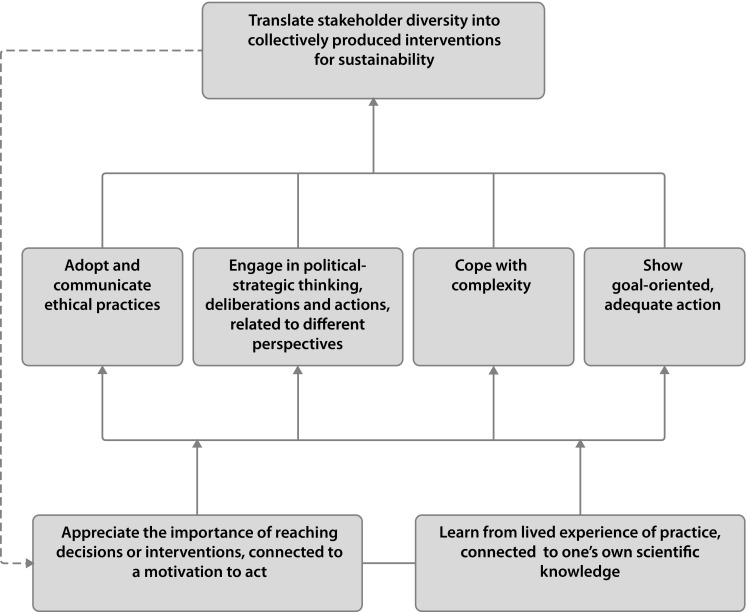
A relational model, showing the dimensions of intervention competence for sustainability. The relations between the dimensions are depicted by lines and the influences by arrows. The dotted arrow illustrates a cyclical process (improving the level of performance)

As became clear during the first workshop, it would be a mistake to consider these dimensions as isolated from one another. If anything, they interrelate, interact and are mutually reinforcing. From this observation, the researchers developed subsequent to the workshops a model showing the inter-relationships which was presented to the participants of both workshops for comment by email. It was confirmed by them as an accurate representation of their deliberations and the final version is shown as Fig. 1 below. This is an influence diagram, where the arrowheads show the direction of influence.

Thus Fig. 1 provides a relational model, corroborated by practitioners, that shows significant complexity, combining the dimensions of intervention competence. Figure 1 takes as its starting point the individual practitioner’s knowledge and learning: the importance of reaching decisions or interventions, and being able to learn from lived experience of practice. Then she/he: (1) engages in political-strategic thinking, deliberations and actions, related to different perspectives; (2) keeps goal-oriented and action-oriented, while adopting and communicating ethical practices during the intervention process. The degree of complexity is handled throughout this process, which requires stakeholder engagement, and finally, he/she is able to translate stakeholder diversity into collectively produced interventions (actions) towards sustainability.

In addition, and also in line with the comments of the professionals on ‘maturity’ and ‘experience’, the dotted arrow at the side of the diagram is meant to illustrate a spiral process, exemplifying that going through the action-learning cycle leads to a higher level of performance. Thus, Fig. 1 may also be used to explain different levels of performance of intervention competence, from basic to excellent. This feedback arrow is a simplified representation of what is undoubtedly a complex process.

In summary, and while by no means representing the last word on the subject, Fig. 1 represents a significant advance on the previous conceptualisation of intervention competence (Perez Salgado et al. 2014): a coherent set of dimensions arises and the results have urged us to introduce a dynamic element by indicating the influences between them.

Our findings are in line with the alumni outcomes on sustainability leadership programmes (MacDonald and Shriberg 2016) in that they emphasise the need for more attention to change-oriented skills, such as conflict resolution, negotiation abilities and public speaking need. Comparison with the quantitative survey of sustainability professionals that was conducted by Willard et al. (2010) is also relevant, since their results contain detailed, quantitative information on several elements of competences. ‘Problem solving’ is rated as the ‘top skill’ by 75% of the respondents, and can be related to four of our dimensions (steer diverse stakeholder perspectives to a solution, cope with complexity, goal-orientedness, and motivation to act), whereby each dimension tackles a specific aspect of ‘problem solving’. Willard et al. (2010) did not investigate, however, the importance of learning from lived experiences and being confident enough in terms of one’s own scientific knowledge to be able to engage with a variety of views that are infused with these non-scientific understandings, whereas this dimension featured strongly in our results and with the practitioners, where it was regarded as fostering creative solutions.

Finally in this section, we address briefly the possible relevance of the presented results for higher education, although this is not a focal point of our article. The results presented here, but also the literature from MacDonald and Shriberg (2016) suggest that ‘doing’ the process towards sustainability is not an easy and straightforward process, and that it contains a diverse and large set of abilities. Our results indicate that these should receive more attention in HE programmes. We referred earlier in the paper to the synthesising work of Wiek et al. (2016, 2011) on sustainability competences. Although this work focuses on sustainability in HE and different HE levels, we note that its designation of strategic (or strategic thinking) and interpersonal/collaboration competences aligns closely with our notion of intervention competence in sustainability professionals. Obviously, there exist possibilities for amalgamation here, or subsuming one competence within another, and we have stated above that we by no means claim our relational model to be definitive. At present, however, in this paper we prefer to continue to base it on ‘intervention competence’. This is because it is grounded in practice and daily experience in ways that the other two are not. It makes sense to practitioners who, in their working lives, have to intervene in a variety of circumstances. A further general challenge to amalgamation is that educationalist perspectives tend to emphasise that competences can be learned, while practitioners will also emphasise more stable, personal traits such as (from Table 1): being inviting, be transparent, show drive, show and practise perseverance.

Also, as reported in the previous section, the need for universalism in competency frameworks has been criticised (Mochizuki and Faveeda 2010). In this regard, we point out that we refer to Fig. 1 above as a ‘model’. It is, however, a distillation of the deliberations of two workshops and therefore is an abstracted, idealised model. We do not expect it to be replicated exactly in practice, but to be used as a starting point for modelling the dynamics of intervention competence in different contexts.

## Discussion: the search for further meaning in the results

*Why* do sustainability professionals think they need the knowledge, skills, attitudes and behaviours that the previous section argues underlie intervention competence? To answer this question we must delve further into the results to search for deeper meaning and interpretation.

The knowledge, skills, attitudes and behaviours that the sustainability professionals identified at the first workshop can be recognised as being necessary for a large part of their jobs which involves working across a variety of boundaries and with multiple stakeholders: within organisations, across organisations, across domains (public, private, NGO), with community groups and the public. As emphasised already in this paper, they are necessary to create the conditions for productive stakeholder engagement. Sometimes they are also necessary for broader institutional change towards sustainability, by which we mean change in organisational values and cultures.

When we examine the words and phrases used by the workshop participants with respect to working across boundaries, it is possible to examine further the nature of achieving effective interventions for sustainability and acquiring intervention competence. Words such as ‘patience’ and ‘perseverance’ which were used during the first workshop suggest not only a drawn-out process in obtaining agreement among the stakeholders, but also a process that is not easy. It very likely involves misunderstanding, disagreement and conflict, where perseverance and patience are necessary in order to progress. Moreover, the need for perseverance and patience indicates that no stakeholder has absolute power to dictate proceedings and that significant time is needed to negotiate the power relations which are at play between stakeholders. Having to operate in a context of conflict was in fact an explicit theme raised by the participants in the second workshop.

In summary, power relations, disagreement and hence conflict between stakeholders comprise a generalised context in which sustainability professionals must operate. Intervention competence, therefore, concerns the anticipation of such conflict and the ability to negotiate it. Thus, expressions in the first workshop of ‘having and showing confidence throughout the process’, ‘have sympathy for all stakeholders’ and ‘be explicit about rewards for different stakeholders’ enable anticipation of areas of disagreement that may turn into conflict. Another phrase used by participants, ‘building trust’, establishes a counter-context for being able to negotiate and minimise disagreement and conflict, while developing ‘joint road maps’ is an essential aspect of such negotiation.

Moreover, disagreement and conflict are not necessarily over technical matters on the best way to do something. While such conflict can, and does, occur from time to time among professionals, the more pervasive and difficult conflicts to negotiate over potential sustainability interventions are likely to be those where the stakeholders have diverse values and non-technical interests in the outcomes. Thus, while the elements for productive engagement almost certainly apply to any professional who has to work in multi-stakeholder contexts, we suggest that this challenge of diverse values and non-technical interests is significantly exacerbated for sustainability professionals—it is endemic to all sustainability-related interventions.

Competing values are particularly difficult to negotiate because of their deontological nature. They lead easily to taking non-negotiable, bottom-line positions. The only practical way forward then is to seek accommodations, meaning agreements and decisions that all of the participating stakeholders may live with, even if they are far from ideal in relation to the underlying values of each (Isaacs, 1993). This, however, is likely to require ‘establishing incentives’—another phrase used by participants at the first workshop—for accommodation. Competing interests may be politically motivated, especially in national strategic and international sustainability issues. Our results show, however, that they may also be local material interests. Although not recorded on the flip-charts that make up Table 1, personal material interests did come to the fore during participant discussions in workshop 1. Examples included: to enhance the budget of one’s unit, to enhance the standing of (or even save) one’s job, to avoid change in one’s well established sustainability practices that will inevitably involve disruption in working lives.

The aim of engagement between stakeholders that is based on communicative action as described in the section “The challenge for productive human engagement in multi-actor settings”, and amply illustrated by the literature review in the section “Professional competences for interventions towards sustainability”, is usually to establish joint interests, or at least introduce measures that will address different interests. Hence, we see in our results of the first workshop, phrases such as: the ability ‘to find common ground’, ‘align conflicting interests’ and ‘build on human similarities’. If this does not work, one has to return to seeking accommodations as the practical way forward. In spite of the above multiple and inter-related challenges of working in multi-stakeholder settings, the workshop participants recognised positive value in exercising patience, perseverance, and building trust. These and related expressions, such as ‘hearing others’ and ‘building bridges’, did not solely concern anticipating conflict and managing it. If done well, multi-stakeholder engagement in their words, has ‘the potential to deliver creativity and broaden perspectives on solutions’, gain ‘buy-in for interventions’, gain ‘local contextual knowledge’, and generally ‘co-create new knowledge’, all of which would be very satisfying personally. In other words, this starts to approach the ‘Habermas’ ideal for communicative action through recognition of the partial nature of one’s own knowledge and hence perspective.

## Conclusions

This article contributes to the field of sustainability science by presenting conceptual ideas and qualitative empirical results regarding competences for sustainability professionals. Sustainability practitioners are important and growing in numbers! They require appropriate skills in order to be effective in interventions and change processes towards sustainability. The empirical results of this paper are based on two workshops within an ongoing action-research programme that enable us to introduce and further analyse the concept of ‘intervention competence for sustainability’. An additional effect of the research is that the results provide practitioners with recognition and credibility for their work and profession.

From the data generated with the professionals, we identified seven dimensions of intervention competence, all of them relevant for obtaining meaningful effective interventions, connected in a relational and dynamic model. Summarising these dimensions as presented in Fig. 1 (section “Results”), intervention competence starts with using ‘one’s lived experience and connecting it to one’s scientific knowledge’ and ‘appreciating the importance of (reaching decisions or interventions’. It ends with the ability to ‘translate stakeholder diversity into collectively produced interventions towards sustainability’. In between the start and end, the following dimensions figure: engaging in political-strategic thinking and actions, related to different perspectives; showing goal-oriented, adequate action; adopting and communicating ethical practices; and coping with the degree of complexity. Via a spiral process different levels of competence may be achieved, from a beginner’s to an expert level.

A key component of this process is engaging with the perspectives of others. It is no surprise that the word ‘perspective’ features prominently in both the sections on results and on ‘search for further meaning’. By promoting the need to understand and take account of different perspectives on a problem, the participating sustainability professionals at least implicitly accepted the limits to their own knowledge. A perspective, therefore, suggests a knowledge boundary and represents an interpretation of the world/phenomena that one wishes to promote, not all possible interpretations.

In the “Introduction” we linked the professionals’ perspective to their lived experience, arguing that the former cannot be reduced to a particular disciplinary, scientific view of a problem. Thus, the diversity of stakeholder views on the nature of the sustainability issue and intervening in it is not simply a diversity of scientific opinions and approaches. As the philosopher Mary Midgley (2014: 6) puts it, ‘(Science) has no private line to reality’. There is a complementary view of the world, also partial but with its own validity, that derives from our lived experiences. We promote in this paper, therefore, a lived experience lens because it lends itself to an understanding of diverse perspectives that goes beyond the need to settle scientific disputes, important as the latter might be. As part of intervention competence, therefore, sustainability professionals need to understand both the contribution of their own lived experiences in forming their perspectives and those of other stakeholders whose perspectives they seek to engage.

The above concluding considerations suggest three further areas of investigation in the ongoing research process:

1. How might intervention competence be acquired (and evaluated)? As sustainability intervention is always a complex process that requires intertwined skills and behaviours, developing these through training is a didactical challenge for trainers, educators and educational scientists. Linking and activating all of the individual dimensions can be done in multiple ways. Most probably, innovative learning models will have to be used. (Examples on pedagogy and evaluation of sustainability competences from the existing literature are Brundiers et al. 2013; Remington-Doucette et al. 2013; Wiek et al. 2016.) It might be more productive in some circumstances to enhance the conditions for informal learning on the job. A more fundamental challenge that was highlighted in the “Results” section, however, concerns how to address the more stable, personal traits that contribute towards competence, and which practitioners will emphasise alongside that which can be learned.
2. Are the inter-related dimensions of intervention competence universal? That is, do they apply equally in all contexts over the world? A further research area, therefore, is to explore intervention competence in Global South and Global North settings. As with all action research approaches, ours’ was conducted in a specific context. A comparative action research approach in different contexts would be a useful way to proceed.

Answering these questions is relevant to sustainability professionals and will contribute to knowledge production in sustainability science, whereby scientists and professionals cooperate together.

## Footnotes

1 Confidentiality: the authors agreed to guard the confidentiality of the input of the participants. This means that the text in Table 1 in the next section has been anonymised, in order to have no relation to specific professionals nor their organisations.
